# Gate-all-around junctionless FET based label-free dielectric/charge modulation detection of SARS-CoV-2 virus†

**DOI:** 10.1039/d1ra08587e

**Published:** 2022-03-23

**Authors:** Kumari Nibha Priyadarshani, Sangeeta Singh, Mustafa K. A. Mohammed

**Affiliations:** Microelectronics & VLSI lab, National Institute of Technology Patna-800005 India nibha@nitp.ac.in sangeeta.singh@nitp.ac.in; Radiology Techniques Department, Dijlah University College Al-Masafi Street Baghdad 00964 Iraq mustafa_kareem97@yahoo.com

## Abstract

The recent corona outbreak has necessitated the development of a label-free, highly sensitive, fast, accurate, and cost-effective biosensor for the detection of SARS-CoV-2 virus. This study records the label-free electrical detection of the SARS-CoV-2 virus using the gate-all-around junctionless field effect transistor (GAA-JLFET) that detects the virus because of the electrical properties (dielectric constant and charge) of spike protein, envelope protein, and virus DNA, for a highly sensitive and real-time bio-sensor. GAA-JLFETs are suitable for this application because of their highest gate controllability, potential vertical stacking, current industry trend compatibility, inherent ease of fabrication, and higher sensitivity. The SARS-CoV-2 virus is first immobilized in the etched nano-cavity embedded beneath the gate electrode, which is then used to detect it. The SARS-CoV-2 virus detection has been calibrated based on the change in system electrical properties after virus immobilization. For effective virus detection, the work takes into account both the dielectric property of S protein and the charge of DNA at the same time. The sensitivity has been calculated using Δ*V*_TH_, Δ*I*_ON_, Δ*g*_m_, and ΔSS. The simulation analysis also shows a simpler recovery mechanism in this case.

## Abstract 2

The present work investigates the label-free electrical detection of the SARS-CoV-2 virus using GAA-JLFET that detects the spike protein, envelope protein, and virus DNA, for a highly sensitive and real-time bio-sensor.

## Graphical abstract

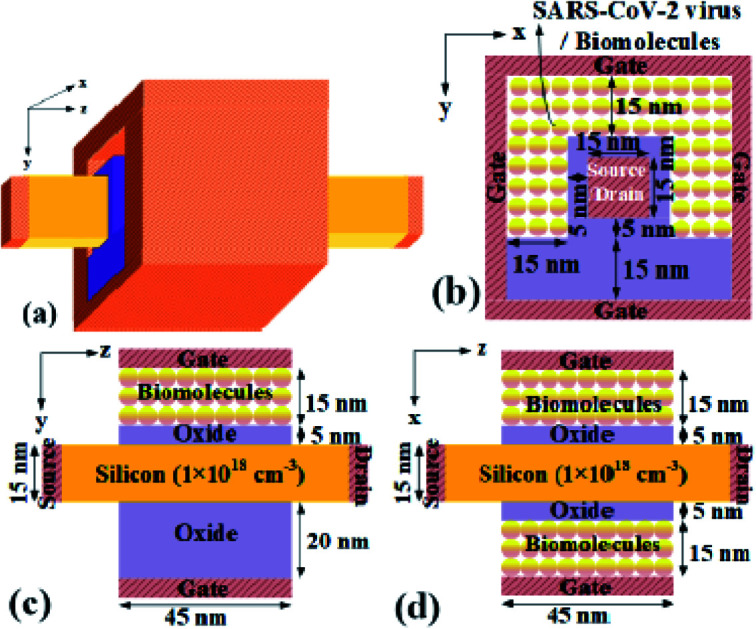

## 1 Introduction

This COVID-19 pandemic has wreaked havoc on society and caused global hysteria. Different zoonotic outbreaks such as Severe Acute Respiratory Syndrome Coronavirus (SARS-CoV) in 2002–2003 (ref. 1) and 2012 the Middle East Respiratory Syndrome Coronavirus (MERS-CoV)^2^ and the current version SARS-CoV-2 are caused by coronavirus only.^3^ The COVID-19's condition was alarming due to its higher transmission rate and mortality rates as compared to the other deadly viral outbreaks.^4–6^ The SARS-CoV-2 virus has a spherical form and a lipid-based envelope membrane. Spike protein (S-protein) protrusions and deposits on the envelope protein (E-protein) and membrane protein (M-protein). A nucleocapsid protein (N-protein) surrounds the RNA, the variation in genome sequence is termed as mutation. It can be the result of genetic copying errors during replication of organisms because of exposure to certain chemicals, ionizing radiations, *etc.* Mutations cause new variations in a species, and cumulative mutations can even lead to the creation of newer species. These mutations could result in virus strains that are more harmful because they are more contagious. The genome of SARS-CoV-2 is made up of more than 30 000 units of ribonucleic acid (RNA). Nucleotides are the building blocks of DNA. Coronaviruses have the largest genome of all the RNA virus families. SARS-CoV-2 virus detection is primarily achieved by two methods. The first is a real-time reverse transcription-Polymerase Chain Reaction (rRT-PCR),^7–10^ and the second is an IgG antibody test using Enzyme Linked Immunosorbent Assays (ELISA). ELISA is a typical procedure for virus detection in which the virus's antibody is found in the patient's blood serum instead of the virus itself.^11^ The second method is based on the rRT-PCR method, in which the kit detects the E-protein gene, RNA (N-protein), open frame reading b1 (OFRb1), and OFRb2 genes in the internal nasal swab.^8^ Viruses mutate while transmitting from one medium to another. Various SARS CoV-2 virus mutant have been identified and labeled by WHO as B.1.1.7 (Alpha), B.1.351 (Beta), B.1.617.2 (Delta), P.1 (Gamma), B.1.526 (Iota), B.1.427 (Epsilon), B.1.429 (Epsilon), B.1.617 (Kappa, Delta). These mutants lead to various repercussions such as high transmission rate, increased morbidity, high number of deaths, mutants are able to bypass the diagnostic tests and are not detected, less susceptible to neutralising antibodies, whether therapeutic or laboratory experiments, mutants are able to bypass natural antibody and causes reinfections, mutants are able to infect even after vaccination, it also increases certain risk such as multi-organ inflammatory syndrome or long COVID, higher affinity to distinct groups, such as children or individuals with compromised immunity.^12^

Detecting the virus and taking precautionary steps are therefore the best measures for the early detection. Furthermore, since there are a limited number of testing kits available and skilled operators are needed, rapid detection of the corona is difficult. At the early stages of virus outbreaks, accurate and rapid diagnosis, as well as successful isolation and care of patients is crucial for the virus containment. This is particularly true when a transmissible disease has no effective cure or fully effective vaccine, as is in this case. Hence, the COVID-19 pandemic has highlighted the importance of leveraging and harnessing our current semiconductor device technology for remote patient detection.^13–15^ We see a need for more robust disease detection for individual and community health, which could be assisted by existing semi-conductor industry growth for the viral tests as the vaccines are slow to emerge for their new variants.

This work reports the label-free electrical detection of the COVID-virus based on S-protein (dielectric constant) and C-DNA (charge density) by deploying the gate-all-around junctionless field effect transistor (GAA-JLFET). Here highly sensitive and real-time bio-sensors are designed. GAA-JLFETs are the most suitable for this application because of their highest gate controllability, potential vertical stacking, current industry trend compatibility, inherent ease of fabrication, and higher sensitivity. The SARS-CoV-2 virus is first immobilized in an engraved nano-cavity embedded beneath the gate electrode, and then it is used to detect it. The identification of the SARS-CoV-2 virus is now calibrated based on the change in system electrical properties following virus immobilization. The study takes into account both the dielectric property of S protein and the charge on the DNA molecule simultaneously for reliable virus detection. The GAA-JLFET structure can be realized using the process suggested in.^16,17^ Further, to realize cavity in the device tunnel-etching process can be used.^18^ In tunnel etching process a SiGe layer is grown on Si substrate and on this layer gate metal contact is grown. This SiGe layer is etched selectively with the help of etchant which has low etching rate and high selectivity for Si, so that it does not damage Si layer. This process needs remote plasma dry etching equipment, which uses fluorocarbon etching gas at high pressure (1500 mT) and low microwave power (200 W).^18–21^

The following is the organization of the work: the device structural parameters, materials, and doping levels are discussed in Section 2. The effect of cavity thickness variation on bio-sensor sensitivity, as well as sensitivity variation with dielectric modulation and charge density modulation is investigated in Section 3. Section 4 brings the research to a conclusion.

## 2 Device structure and simulation models

**Fig. 1:**
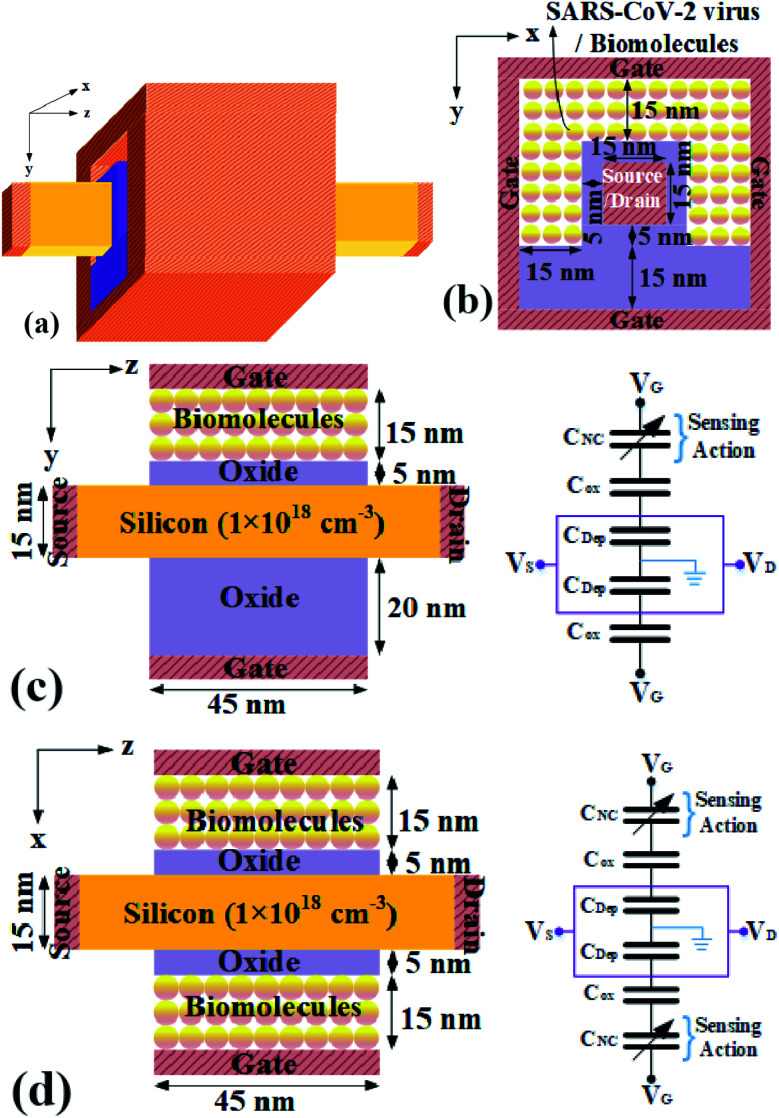
GAA-JLFET device structure for SARS-CoV-2 virus detection (a) 3D-view and 2-D cross-sectional view along planes, (b) the *x*–*y*, (c) the *z*–*y* and its capacitance model to illustrate sensing, and (d) the *z*–*x* and its capacitance model to illustrate the biosensing.

Gate-all-around Junctionless FET (GAA-JLFET) based SARS-CoV-2 virus sensor has been designed because of its single uniform doping, thus easy fabrication and cost-effectiveness. The 3D structure of the GAA-JLFET sensor, 2D cross-sectional view along the *x*–*y* plane, *z*–*y* plane, *z*–*x* plane are shown in Fig. 1(a), (b), (c) and (d), respectively along with its capacitance model for the device in Fig. 1(c) and (d), where *C*_NC_ shows the capacitance of the nano-cavity, where *C*_ox_ stands for the capacitance of the oxide layer, and where *C*_Dep_ shows the depletion capacitance of the device. The capacitance model of the device illustrates that the variation in the electrical property of the virus immobilized in the cavity will vary the capacitance of the nano-cavity and this will lead to the change in the device characteristics sensing the virus. The gate length is kept in accordance with the state-of-art technology *i.e.* 45 nm with work-function of gate electrode considered as 5.93 eV (platinum, Pt). All the device structural parameters are shown in Fig. 1. The doping of silicon region is kept as 1 × 10^18^ cm^−3^ of n-type. The cavity region is 15 nm thick under three gates for the immobilization of bio-molecules. Bio-molecule adsorption test is performed during fabrication of sensor by keeping bio-molecule in the cavity till equilibrium between the adsorbed bio-molecule and the bio-molecule in the cavity is reached. A uniform temperature of 25 °C is kept using a thermostat shaker bath. The concentration of bio-molecule present in the solution before and after adsorption is used to calculate the amount of bio-molecule adsorbed. Further, a 5 nm thick SiO_2_ layer is used for the enhanced binding of the bio-molecules. The study is done by deploying technology computer aided design (TCAD) tool Silvaco ATLAS.^22^ The drift-diffusion transport model (cvt) is used to model the carrier transport. Concentration dependent and field dependent mobility models are used along with SRH and auger recombination for the precise modeling of carrier recombination. Band gap narrowing model (BGN) is also used. Gummel and newton trap numerical solvers have been used to improve the convergence. Here, the basic bio-molecule detection mechanism is based on the dielectric and charge based modulation effect due to the SARS-CoV-2 virus immobilization in the nano-cavity embedded below the gate electrode.^23–27^

## 3 Result and discussion

The dielectric constant of protein protrusions of SARS-CoV-2 virus varies between 2 to 4.^13–15,28–31^ This property can be utilized for the electrical detection of the virus. Further, the presence of charge density in the DNA of the bio-molecule also provides scope for virus detection based on charge density. In this work, we have studied the detection of viruses based on dielectric modulation as well as charge modulation.^32–36^

### 3.1 Sensitivity analysis of S-protein dielectric modulation

**Fig. 2:**
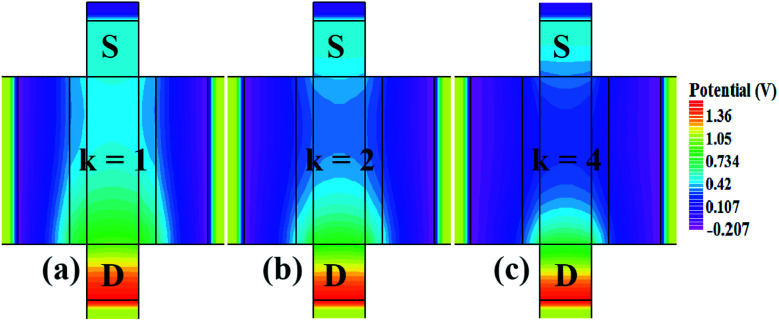
Potential contour for neutral bio-molecule with (a) *κ* = 1 (air) in the cavity, (b) *κ* = 2 in the cavity, and (c) *κ* = 4 in the cavity at *V*_GS_ = 1 V and *V*_DS_ = 1 V.

**Fig. 3:**
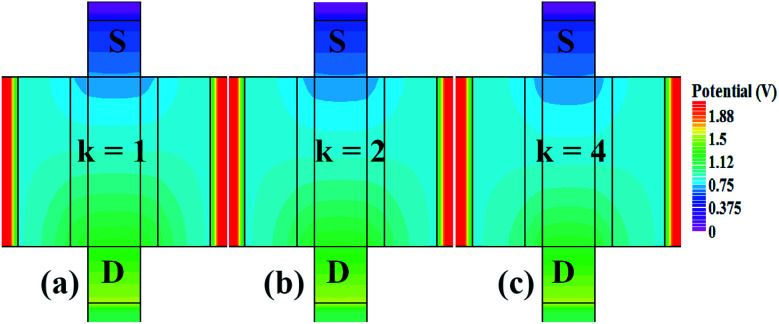
Potential contour for neutral bio-molecule with (a) *κ* = 1 (air) in the cavity, (b) *κ* = 2 in the cavity, and (c) *κ* = 4 in the cavity at *V*_GS_ = 2 V and *V*_DS_ = 1 V.

**Fig. 4:**
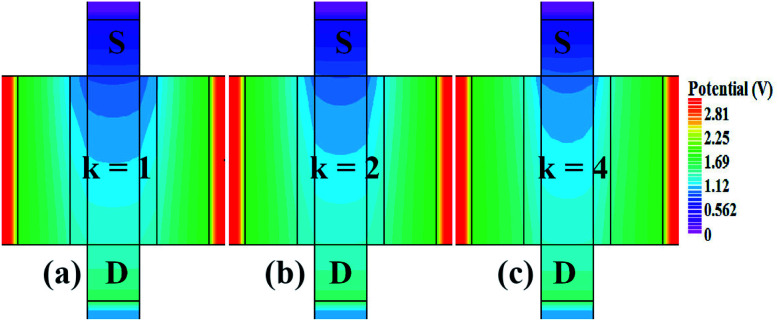
Potential contour for neutral bio-molecule with (a) *κ* = 1 (air) in the cavity, (b) *κ* = 2 in the cavity, and (c) *κ* = 4 in the cavity at *V*_GS_ = 3 V and *V*_DS_ = 1 V.

**Fig. 5:**
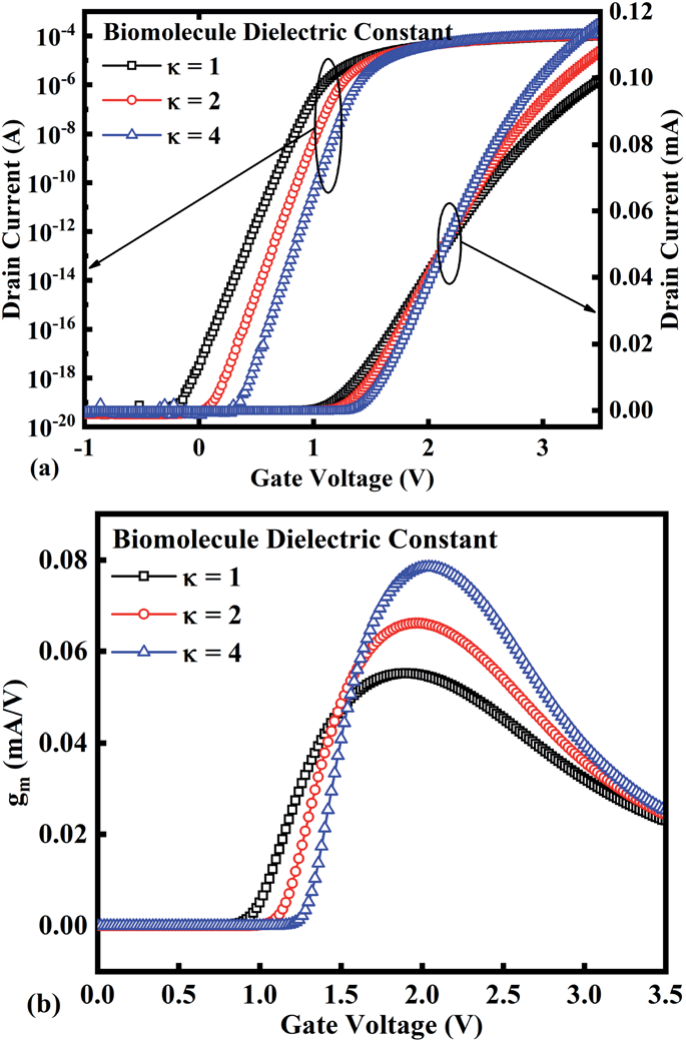
(a) Transfer characteristics and (b) transconductance at *V*_DS_ = 1 V for the bio-molecules with dielectric constant variations.

**Fig. 6:**
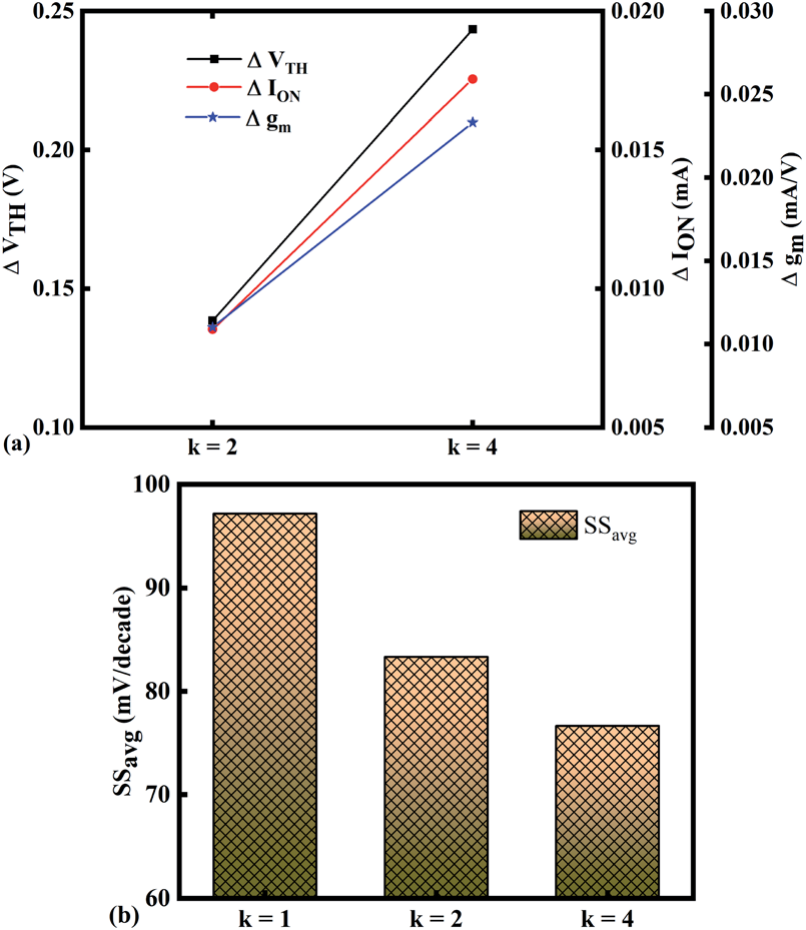
Sensitivity of bio-molecules with dielectric constant variation in terms of (a) Δ*V*_TH_, Δ*I*_ON_, and Δ*g*_m_ with reference to air in the cavity and (b) variation in SS_avg_ for bio-molecules with dielectric constant variations.

In our analysis, we have taken the dielectric constant of SARS-CoV-2 virus as 2 and 4. Fig. 2 shows the potential contour for GAA-JLFET SARS-CoV-2 sensor *x*–*z* cross-section for the dielectric constant of bio-molecule in the cavity is taken as 1 (air), 2 and 4 for *V*_GS_ = 1 V and *V*_DS_ = 1 V. Fig. 3 shows the potential contour for GAA-JLFET SARS-CoV-2 sensor *x*–*z* cross-section for the dielectric constant of bio-molecule in cavity is taken as 1 (air), 2 and 4 for *V*_GS_ = 2 V and *V*_DS_ = 1 V and Fig. 4 shows the potential contour for GAA-JLFET SARS-CoV-2 sensor *x*–*z* cross-section for the dielectric constant of bio-molecule in the cavity is taken as 1 (air), 2, and 4 for *V*_GS_ = 3 V and *V*_DS_ = 1 V. It is observed from the contour plot in Fig. 2 that potential in the device decreases with an increase in the dielectric constant of bio-molecule and in Fig. 3 this difference in potentials decreases with an increase in dielectric constant. Further, in Fig. 4 the potential slightly increases with an increase in the dielectric constant. The surface potential decreases with an increase in the dielectric constant for *V*_GS_ = 1 V leading to a decrease in drain current and increase in threshold voltage with an increase in the dielectric constant. The decrease in surface potential with an increase in dielectric constant is minimized for *V*_GS_ = 2 V thus the decrease in drain current with an increase in dielectric constant reduces. Further, the surface potential increases with an increase in dielectric constant for *V*_GS_ = 3 V leading to increase in drain current with increase in dielectric constant. This drain current behaviour with respect to gate voltage/transfer characteristics are shown in Fig. 5(a) for the variation in the dielectric constant of bio-molecule in the cavity. The threshold voltage of the sensor increases with an increase in the dielectric constant of bio-molecule in the cavity of the sensor. Fig. 5(b) shows the transconductance of the GAA-JLFET sensor with the dielectric constant variation of the bio-molecule. The peak transconductance increases with an increase in the dielectric constant. Here, *V*_TH_, *I*_ON_, *I*_ON_/*I*_OFF_, *g*_m_ and SS_avg_ of GAA-JLFET sensor with the dielectric constant variation of bio-molecule are listed in Table 1. Further, Δ*V*_TH_, Δ*I*_ON_, and Δ*g*_m_ are shown in Fig. 6(a) where Δ*V*_TH_, Δ*I*_ON_ and Δ*g*_m_ represent the change in *V*_TH_, *I*_ON_ and *g*_m_ with respect to air in the cavity, respectively and is given as

1 Δ*V*_TH_ = |*V*_TH,*κ*_ − *V*_TH,air_|

2 Δ*I*_ON_ = |*I*_ON,*κ*_ − *I*_ON,air_|

3 Δ*g*_m_ = |*g*_m,*κ*_ − *g*_m,air_|

**Table 1:** GAA-JLFET sensor parameters variation with dielectric modulation and charge modulation

	*V*_TH_ (V)	*I*_ON_ (A)	*I*_ON_/*I*_OFF_	*g*_m_ (A/V)	SS_avg_ (mV per decade)
Dielectric modulation					
Air	1.25352	9.9496 × 10^−5^	3.67972 × 10^15^	5.51979 × 10^−5^	97.166
*κ* = 2	1.39199	1.08027 × 10^−4^	3.95461 × 10^15^	6.62049 × 10^−5^	83.333
*κ* = 4	1.9701	1.17048 × 10^−4^	4.27362 × 10^15^	7.85259 × 10^−5^	76.666
Charge modulation					
− 1 × 10^12^	3.18357	1.35358 × 10^−5^	8.49015 × 10^14^	4.2777 × 10^−5^	90
− 5 × 10^11^	2.2574	6.39758 × 10^−5^	2.02474 × 10^15^	5.35296 × 10^−5^	93.33
− 1 × 10^11^	1.45627	9.394 × 10^−5^	2.8792 × 10^15^	5.5072 × 10^−5^	95.66
0	1.25352	9.9496 × 10^−5^	3.67972 × 10^15^	5.51979 × 10^−5^	97.166
1 × 10^11^	1.04988	1.04461 × 10^−4^	9.1356 × 10^15^	5.5294 × 10^−5^	98.333
5 × 10^11^	0.22648	1.19824 × 10^−4^	4.7168 × 10^15^	5.51121 × 10^−5^	111.666
1 × 10^12^	−0.81903	1.32565 × 10^−4^	2.46308 × 10^15^	5.35667 × 10^−5^	130

The SS_avg_ of the sensor also varies with the dielectric constant variation of the bio-molecules. The SS_avg_ of the sensor improves with an increase in the dielectric constant of bio-molecule. Here, SS_avg_ is defined as4
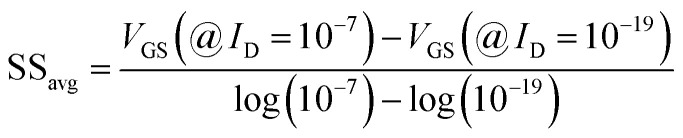


The SS_avg_ plot is shown in Fig. 6(b). Here, Δ*V*_TH_ is in the range of 0.13 V and 0.24 V, Δ*I*_ON_ is in the range of 10^−6^ A and Δ*g*_m_ is in the range of 10^−6^ A/V, also the variation of SS_avg_ with variation in dielectric constant ensures good sensitivity with dielectric modulation and thus precise detection of bio-molecule/virus.

### 3.2 Sensitivity analysis of DNA charge density modulation

The presence of charge density in the DNA of the virus makes it traceable with charge density modulation. The interface trap charge density considered for the study is − 1 × 10^12^ cm^−2^ to 1 × 10^12^ cm^−2^ with a fixed dielectric constant of bio-molecule, the dielectric constant is 1.

**Fig. 7:**
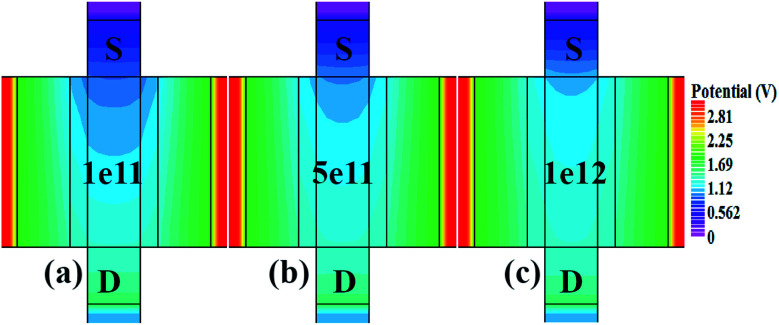
Potential contour for positive charge density variations of bio-molecule in cavity with (a) *N*_f_ = 1 × 10^11^, (b) *N*_f_ = 5 × 1^11^, and (c) *N*_f_ = 1 × 1^12^ at *V*_DS_ = 1 V and *V*_GS_ = 3 V.

**Fig. 8:**
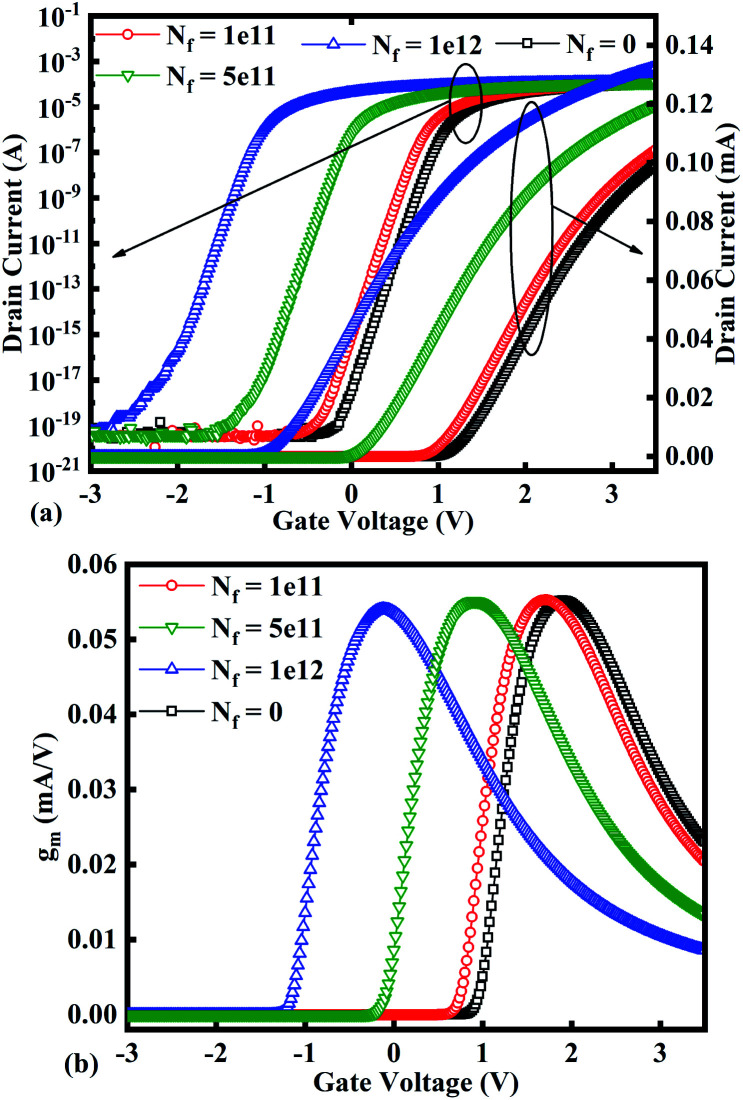
(a) Transfer characteristics and (b) transconductance at *V*_DS_ = 1 V for different positive bio-molecules charges densities.

**Fig. 9:**
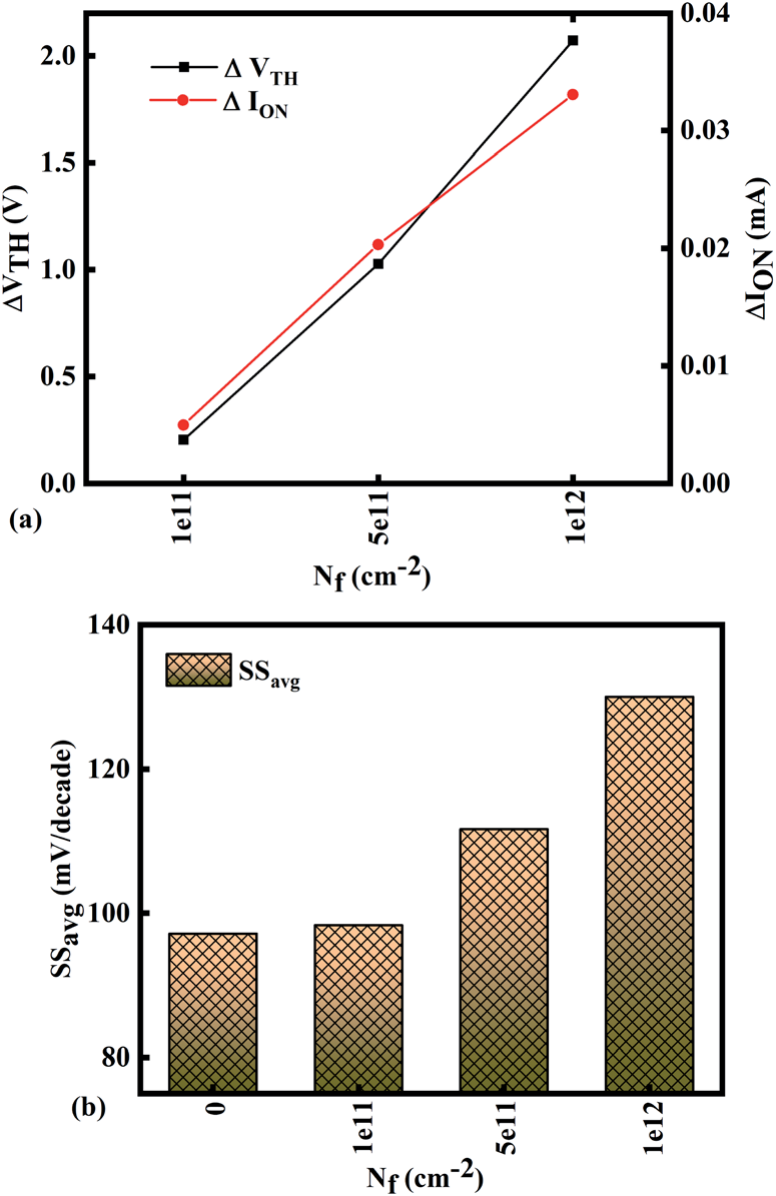
Sensitivity for different positive bio-molecules charge density in cavity in terms of (a) Δ*V*_TH_ and Δ*I*_ON_ with reference to neutral bio-molecule in the cavity and (b) variation in SS_avg_ for different positive bio-molecule charge densities.

Fig. 7(a)–(c) show the potential contours for GAA-JLFET *x*–*z* cross-section with bio-molecule charge density taken as 1 × 10^11^ cm^−2^, 5 × 10^11^ cm^−2^, and 1 × 10^12^ cm^−2^, respectively at *V*_GS_ = 3 V and *V*_DS_ = 1 V. The flat band voltage decreases with an increase in positive charge (
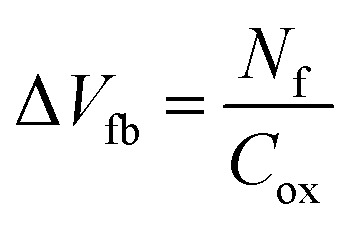
, where Δ*V*_fb_ is the change in flat band voltage, *N*_f_ is the concentration of trap charge and *C*_ox_ is oxide capacitance) and thus it leads to an increase in the effective potential (*V*_eff_ = *V*_GS_ − *V*_fb_, where *V*_eff_ is the effective potential at surface and *V*_GS_ is gate to source supply voltage).^37^ The potential contour shows an increase in potential with increase in the positive charge density of bio-molecules. The increase in surface potential with an increase in the positive charge density leads to an increase in the drain current and thus a decrease in threshold voltage as shown in Fig. 8(a). Fig. 8(b) shows the transconductance of GAA-JLFET sensor with the positive charge density variation of bio-molecules. The peak transconductance remains similar with the variation in *V*_GS_ for peak transconductance with an increase in positive charge density. The increase in the positive charge density leads to a decrease in *V*_GS_ value for peak transconductance. The *V*_TH_, *I*_ON_, *I*_ON_/*I*_OFF_, *g*_m_, and SS_avg_ of GAA-JLFET sensor with the positive charge density variation of bio-molecule is listed in Table 1. Further, Δ*V*_TH_ and Δ*I*_ON_ is shown in Fig. 9(a) where Δ*V*_TH_ and Δ*I*_ON_ denote the change in *V*_TH_ and *I*_ON_ with respect to the neutral bio-molecule in cavity, respectively and is given as

5 Δ*V*_TH_ = |*V*_TH,charge_ − *V*_TH,neutral_|

6 Δ*I*_ON_ = |*I*_ON,charge_ − *I*_ON,neutral_|

The SS_avg_ of the sensor also varies with the positive charge density variation of bio-molecule. The SS_avg_ of the sensor degrades with an increase in the positive charge density of bio-molecule. The SS_avg_ plot is shown in Fig. 9(b). The Δ*V*_TH_ is in the range of 1–2 V and Δ*I*_ON_ is in the range of 10^−5^ A, also the variation of SS_avg_ with variation in positive charge density ensures good sensitivity with positive charge density and thus precise detection of virus.

**Fig. 10:**
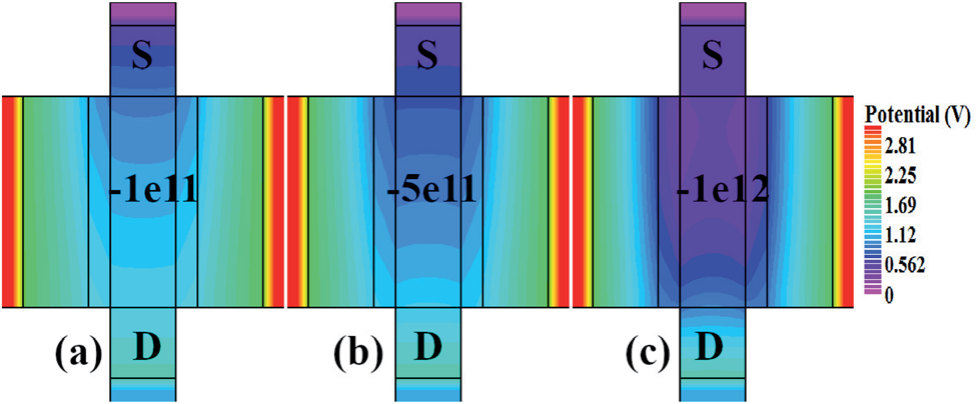
Potential contour for negative charge density variation of bio-molecule in cavity with (a) *N*_f_ = − 1 × 10^11^, (b) *N*_f_ = − 5 × 1^11^, and (c) *N*_f_ = − 1 × 1^12^ at *V*_DS_ = 1 V and *V*_GS_ = 3 V.

**Fig. 11:**
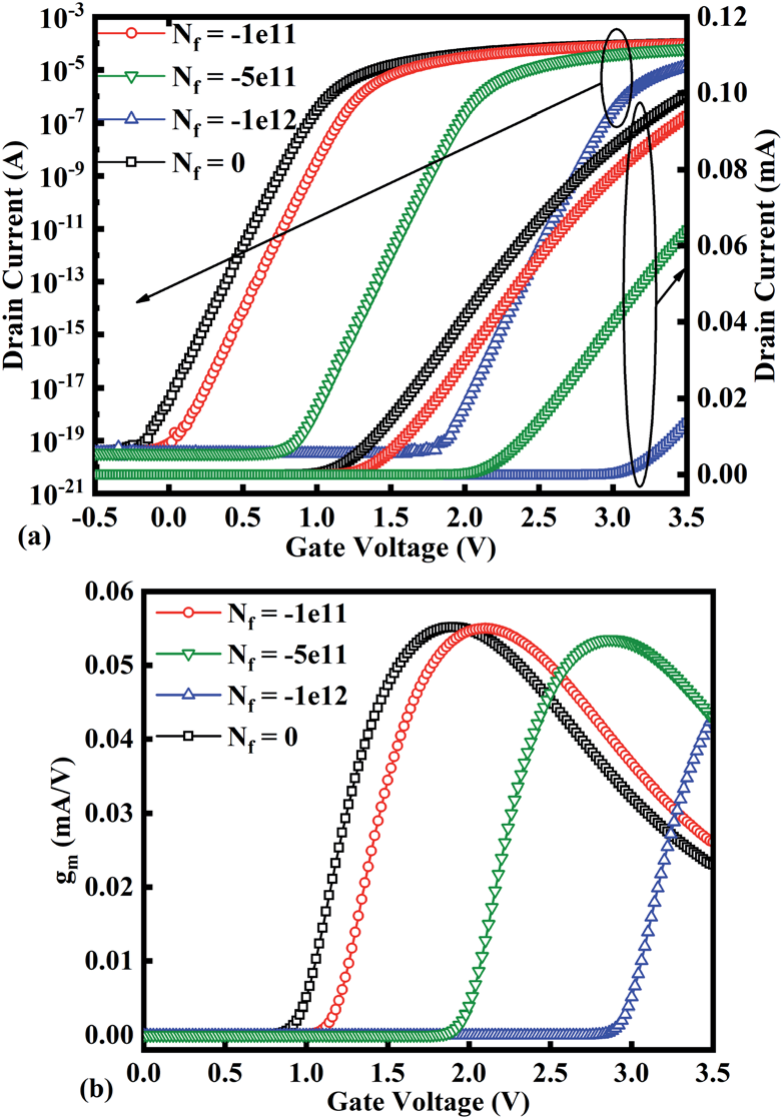
(a) Transfer characteristics and (b) transconductance at *V*_DS_ = 1 V for different negative bio-molecules charge densities.

**Fig. 12:**
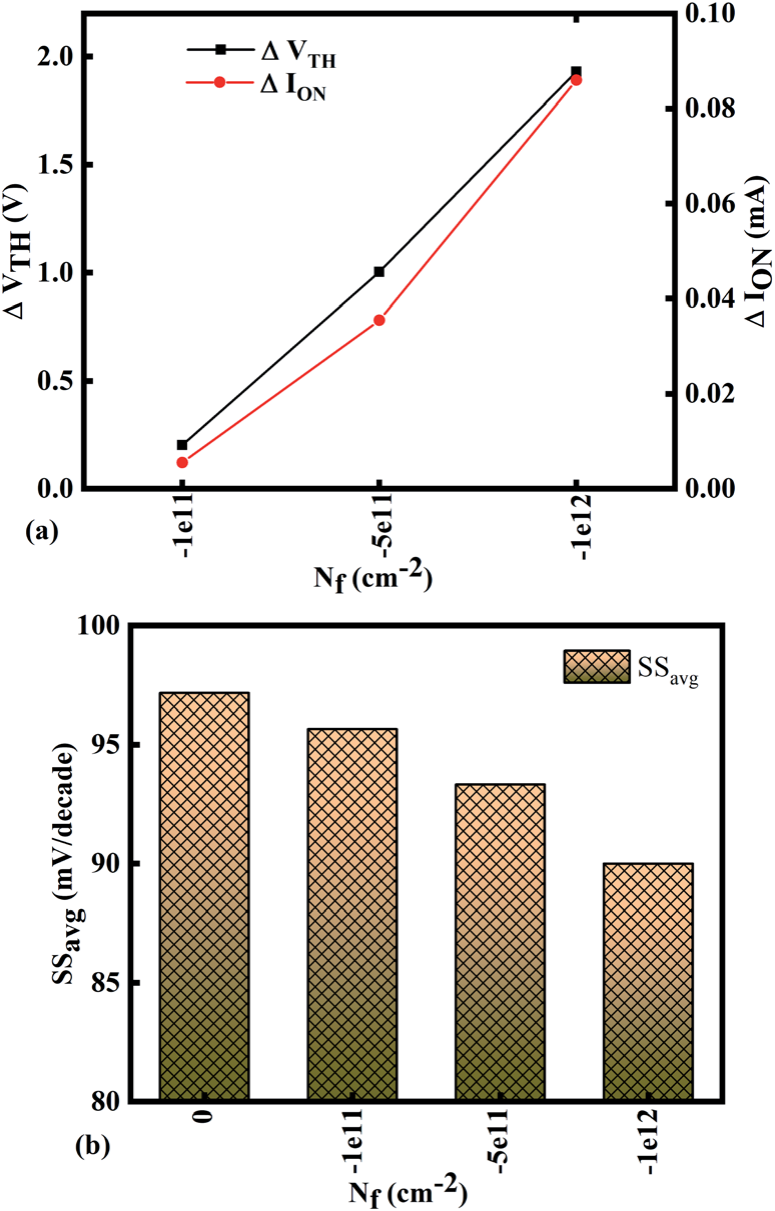
Sensitivity for different negative bio-molecules charge densities in cavity in terms of (a) Δ*V*_TH_ and Δ*I*_ON_ with reference to neutral bio-molecule in the cavity and (b) variation in SS_avg_ for different negative bio-molecule charge densities.

Fig. 10(a)–(c) show potential contour for GAA-JLFET *x*–*z* cross-section with bio-molecule charge density is considered as −1 × 10^11^ cm^−2^, −5 × 10^11^ cm^−2^, and −1 × 10^12^ cm^−2^ respectively at *V*_GS_ = 3 V and *V*_DS_ = 1 V. The flat band voltage increases with an increase in negative charge and thus it leads to decrease in effective potential.^37^ The potential contour shows a decrease in potential with an increase in negative charge density of the bio-molecules. The decrease in surface potential with an increase in the negative charge density leads to a decrease in the drain current and thus an increase in threshold voltage as shown in Fig. 11(a). Fig. 11(b) shows the transconductance of GAA-JLFET sensor with negative charge density variation of bio-molecules. The peak transconductance remains similar to the variation in *V*_GS_ for peak transconductance with an increase in negative charge density. The increase in negative charge density leads to an increase in *V*_GS_ value for peak transconductance. The *V*_TH_, *I*_ON_, *I*_ON_/*I*_OFF_, *g*_m_, and SS_avg_ of GAA-JLFET sensor with the negative charge density variation of bio-molecule are listed in Table 1. Further, Δ*V*_TH_ and Δ*I*_ON_ are shown in Fig. 12(a) where Δ*V*_TH_ and Δ*I*_ON_ represent the change in *V*_TH_ and *I*_ON_ with respect to neutral bio-molecule in the cavity, respectively and as given by eqn (5) and (6). The SS_avg_ of the sensor also varies with the negative charge density variation of bio-molecule. The SS_avg_ of the sensor improves with an increase in negative charge density of bio-molecule. The SS_avg_ plot is shown in Fig. 12(b). The Δ*V*_TH_ is in the range of 1–2 V and Δ*I*_ON_ in the range of 10^−5^ A, also the variation of SS_avg_ with variation in negative charge density ensures good sensitivity with negative charge density and thus precise detection of virus.

## 4 Conclusion

This work reports the label-free electrical detection of SARS-CoV-2 virus using the gate-all-around junctionless field effect transistor (GAA-JLFET). The study takes into account simultaneously both the dielectric property of S protein and the charge of DNA for detection of the SARS-CoV-2 virus. The sensitivity has been analyzed in terms of Δ*V*_TH_, Δ*I*_ON_, Δ*g*_m_ and SS. This higher variation in these electrical parameters helps in realizing highly sensitive biosensors. GAA-JLFET is deployed here owing to its easy fabrication and higher sensitivity due to higher gate controllability with the increased number of gates. The basic mechanism for SARS-CoV-2 virus detection is the immobilization of the virus in the etched nano-cavity embedded beneath the gate electrode. Further, the change in the device's electrical properties post virus immobilization is calibrated for the SARS-CoV-2 virus detection. This study shows very high sensitivity towards DNA charge density. The TCAD investigation pledges for the potentials of this structure for the array-based screening and *in vivo* bio-species diagnostics.

## Conflicts of interest

There are no conflicts to declare.

## Supplementary Material

RA-012-D1RA08587E-s001
