# Supplementary material for: Gate-all-around junctionless FET based label-free dielectric/charge modulation detection of SARS-CoV-2 virus
Source: RSC Adv. 2022 Mar 23;12(15):9202–9. doi: 10.1039/d1ra08587e (PMC8985138; doi:10.1039/d1ra08587e)
Supplement: RA-012-D1RA08587E-s001 [file RA-012-D1RA08587E-s001.pdf]

Electronic Supplementary Material (ESI) for RSC Advances.
This journal is © The Royal Society of Chemistry 2022

Supporting Information

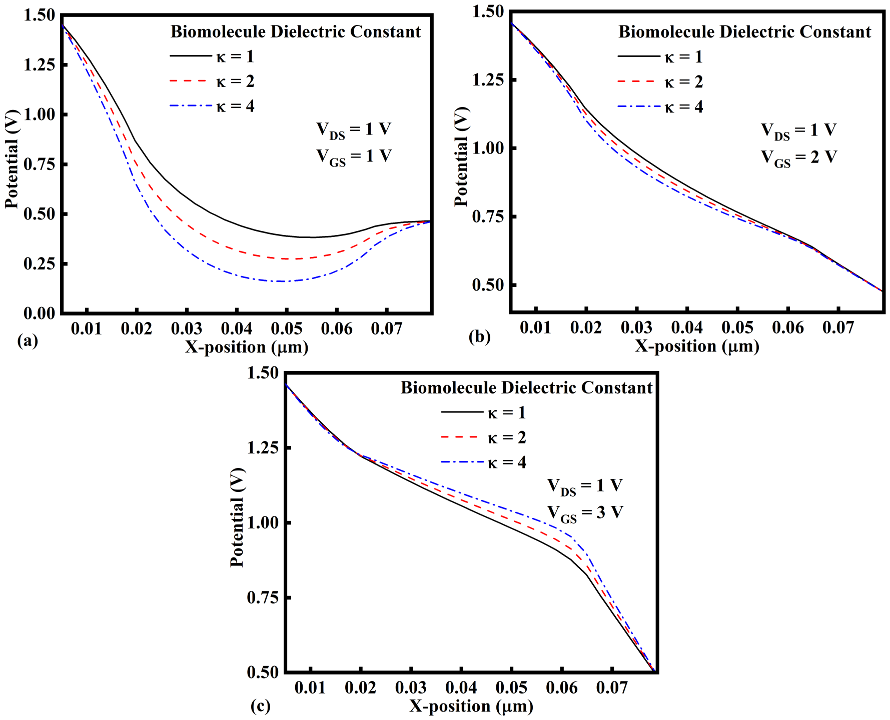

Fig. 1 Surface potential along channel length for bio-molecule with dielectric constant variation at V_{DS} = 1 V and (a) V_{GS} = 1 V, (b) V_{GS} = 2 V and (c) V_{GS} = 3 V.

The variation in surface potential of the device for bio-molecule with dielectric constant variation is shown in Fig. 1 (a), (b) and (c) for V_{GS} = 1 V, V_{GS} = 2 V and V_{GS} = 3V respectively with V_{DS} = 1 V. The surface potential decreases with an increase in the dielectric constant for V_{GS} = 1 V leading to a decrease in drain current and an increase in the threshold voltage with an increase in the dielectric constant. The decrease in surface potential with an increase in dielectric constant is minimized for V_{GS} = 2 V thus the decrease in drain current with an increase in dielectric constant reduces. Further, the surface potential increases with an increase in dielectric constant for V_{GS} = 3 V leading to an increase in the drain current with an increase in the dielectric constant.

The potential contour shows an increase in potential with increase in positive charge density of bio-molecules, which is clearly shown in surface potential plot in Fig. 2. The increase in surface potential with an increase in positive charge density leads to an increase in drain current and thus a decrease in threshold voltage. The potential contour shows a decrease in potential with an increase in negative charge density of bio-molecules, which is clearly shown in the surface potential plot in Fig. 3. The decrease in surface potential with an increase in the negative charge

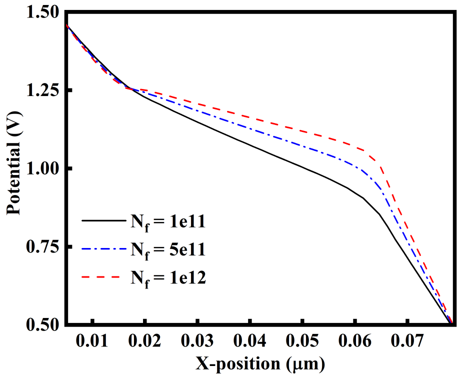

Fig. 2 Surface potential variation along with the channel-length for different positive N_f values at $V_{DS} = 1$ V and $V_{GS} = 3$ V.

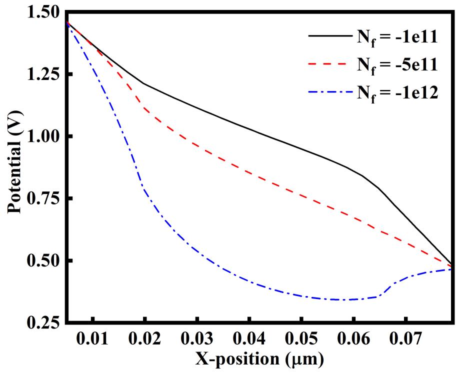

Fig. 3 Surface potential variation along with the channel-length for different negative N_f values at $V_{DS} = 1$ V and $V_{GS} = 3$ V.

density leads to a decrease in the drain current and thus an increase in threshold voltage.
